# Effectiveness of Hyaluronic Acid Administration in Treating Adhesive Capsulitis of the Shoulder: A Systematic Review of Randomized Controlled Trials

**DOI:** 10.1155/2015/314120

**Published:** 2015-01-31

**Authors:** Lin-Chien Lee, Fu-Kong Lieu, Hung-Lin Lee, Tao-Hsin Tung

**Affiliations:** ^1^Department of Physical Medicine and Rehabilitation, Cheng Hsin General Hospital, Taipei 11220, Taiwan; ^2^Department of Nursing, College of Medicine, National Taiwan University, Taipei 10051, Taiwan; ^3^Department of Medical Research and Education, Cheng Hsin General Hospital, Taipei 11220, Taiwan; ^4^Department of Public Health, School of Medicine, Fu Jen Catholic University, Taipei 24205, Taiwan

## Abstract

*Introduction*. Adhesive capsulitis (AC) of the shoulder presents with an insidious onset of pain and progressive limitation of shoulder movement. *Objectives*. To investigate whether intra-articular hyaluronic acid (HA) administration alone is superior to conventional therapies and whether the addition of intra-articular HA administration to conventional therapies improves clinical outcomes in patients with AC. *Methods*. The PubMed, EMBASE, CINAHL, and Cochrane Library electronic databases were searched without language restrictions in July 2014 with *a priori* defined inclusion and exclusion criteria. *Results*. Four randomized controlled trials (273 participants, 278 shoulders) were included in this review. Two trials compared intra-articular HA administration with conventional therapies and 2 trials evaluated intra-articular HA administration as an addition to conventional therapies. Pain and shoulder function/disability outcomes in the HA injection group were not superior to those achieved in the conventional therapy groups. No significant differences in pain or shoulder function/disability outcomes were noted between the groups with and without adjunctive HA administration. *Conclusions*. Intra-articular HA administration alone is not superior to conventional AC treatments, and the addition of intra-articular HA administration to conventional therapies does not provide significant added benefits. HA administration in AC patients who are receiving conventional therapies should be evaluated to avoid unnecessary medical expenditure.

## 1. Introduction

Adhesive capsulitis (AC) of the shoulder manifests as an insidious onset of shoulder pain and the progressive limitation of active and passive shoulder movement, resulting in a marked disability and difficulty with daily activities. AC occurs in 2–5% of the general population, and predominantly in females. The age of onset of AC ranges from 40 to 60 years. The etiology and pathogenesis of primary AC remain largely unknown, but idiopathic inflammation in the lining of the shoulder joint is postulated to be the primary cause [1, 2]. AC is associated with a variety of diseases, including diabetes mellitus, thyroid dysfunction, and autoimmune diseases, as well as a history of breast cancer treatment, stroke, or myocardial infarction [3]. The natural course of primary AC is not well established, and the clinical diagnosis is based on patient history and physical examination [4].

Treatment of AC aims to relieve pain and restore shoulder motion and function. Conventional therapies for AC include the use of analgesics and nonsteroidal anti-inflammatory drugs, intra-articular corticosteroid administration, and physical therapy [4]. Treatment programs for AC usually combine a number of the aforementioned modalities to address the various symptoms and to achieve maximal outcomes [5]. In patients refractory to conventional therapies, more aggressive interventions such as capsular distention, manipulation under anesthesia, and surgery have been used [3].

Hyaluronic acid (HA) is an integral component of the synovial fluid and is crucial for the lubrication and chondroprotection of joints. By injecting HA into joints, cytokine-induced responses are suppressed and synovial inflammation is reduced, alleviating pain and improving joint function [6, 7]. Furthermore, when HA is injected intra-articularly to treat AC, the degree of suppressed inflammation is directly associated with the improvement in shoulder function [8]. The effectiveness of viscosupplementation with HA injection has been confirmed in the treatment of knee osteoarthritis [9, 10]. However, the utility of HA administration in the treatment of AC is not universally endorsed, because of controversial evidence [11–13]. The objectives of our review were to investigate whether HA administration alone is superior to conventional AC therapies (e.g., intra-articular corticosteroid injection and physical therapy) and whether the addition of HA administration to conventional therapy regimens improves clinical outcomes in patients with AC.

## 2. Methods

### 2.1. Review Protocol

Our methodology and reporting were guided by the Preferred Reporting Items for Systematic Reviews and Meta-Analyses (PRISMA) statement and checklist [14]. This systematic review was accepted by the online PROSPERO international prospective register of systematic reviews of the National Institute for Health Research (CRD42014010363).

### 2.2. Data Sources and Searches

We systematically searched PubMed, EMBASE, CINAHL, and the Cochrane Library without language restrictions in July 2014. We entered “adhesive capsulitis,” “frozen shoulder,” “stiff shoulder,” “periarthritis,” “Duplay's disease,” “hyaluronic acid,” “sodium hyaluronate,” and “viscosupplementation” as keywords for searches. We also searched reference lists of retrieved trials and contacted known experts in the field for potentially relevant studies not identified by the internet-based search. Unpublished trials were searched using the ClinicalTrials.gov registry (https://clinicaltrials.gov/).

### 2.3. Study Selection

Randomized controlled trials that directly compared HA injection (into the glenohumeral joint or the subacromial bursa) with conventional therapies (intra-articular corticosteroid injection or physical therapy) or that investigated the effectiveness of HA administration as an added treatment were eligible for inclusion. Prospective uncontrolled studies were excluded. Included trials must have clearly documented the inclusion and exclusion criteria for patient selection and information regarding HA administration. The symptoms of AC (pain, limited range of motion (ROM), and disability of the shoulder) must have persisted in patients for more than 1 month before trial registration. The diagnosis of AC must have been confirmed by clinical and/or ultrasonographic assessments. Quantitative assessments of shoulder pain, ROM, or function/disability before and following HA injections were reported in the included trials. A minimum of 1 month of follow-up was required. Trials that evaluated the treatment of causes of shoulder pain other than primary AC were excluded. When papers with overlapping data sets were published, the trial with the larger population was included.

### 2.4. Data Extraction and Quality Assessment

One author (Lin-Chien Lee) evaluated all potential studies eligible for inclusion and used a standardized form to extract data, including study population characteristics, study design, inclusion and exclusion criteria, information regarding HA administration, outcome measurements, and frequency of adverse events. A second author (Fu-Kong Lieu) verified the accuracy and completeness of the extracted data. The authors of included trials were contacted if additional information was necessary. Two authors (Lin-Chien Lee and Fu-Kong Lieu) independently evaluated the methodological quality of the included studies. The following domains were assessed: allocation generation and concealment, blinding, duration of follow-up, numbers of subjects not included in follow-up, and data analysis method (intention-to-treat or per protocol). Discrepancies were resolved by discussion and consensus.

### 2.5. Data Synthesis and Analysis

The outcomes of effectiveness were protocol-defined pain score, ROM, and shoulder function/disability scores at postinjection follow-up assessments more than 1 month after administration. Adverse events were also a main outcome of interest. The included trials were summarized qualitatively. We quantitatively combined trials using meta-analysis if the study designs were sufficiently similar with regard to the study population, interventions being compared, and measured outcomes.

## 3. Results

### 3.1. Characteristics of the Included Trials

**Figure 1:**
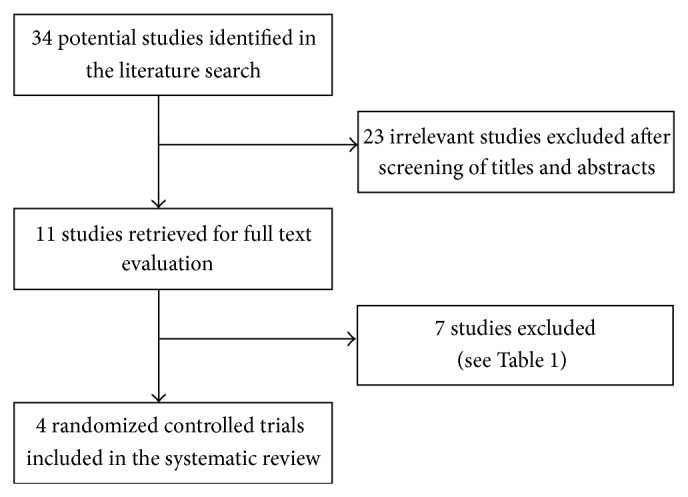
Flow diagram of the evaluation process for the studies included in the systematic review.

Thirty-four nonduplicate studies were identified in the literature search, and 11 full text reports of clinical trials were retrieved and screened for eligibility (Figure 1). One observational study not involving treatment [15] and 3 prospective uncontrolled studies were excluded [8, 16, 17]. One study was excluded because of a postinjection follow-up interval shorter than 1 month (7 days) [18]. One study was excluded because the study participants included shoulder osteoarthritis and rotator cuff tear patients in addition to those with AC [19]. One study was excluded because the authors combined corticosteroid and HA injections in the treatment course (1 dose of corticosteroid followed by 5 weekly doses of HA), and pain reduction effects and functional improvements could have been caused by the corticosteroid, HA, or both [20]. Detailed reasons for the exclusion of studies are summarized in Table 1.

Four trials with variable methodological quality met our inclusion criteria, all of which had been published as peer-reviewed articles. Study population characteristics and experimental design information for each of the 4 trials included in this review are shown in Table 2. The number of participants in the included trials ranged from 30 to 90, and 273 participants (278 shoulders) were included in our systematic review. Mean ages ranged from 54.5 to 64.2 years. The duration from the onset of AC symptoms to trial registration ranged from 1 to 8.3 months. Musculoskeletal ultrasonography was used to confirm the diagnosis of AC in 2 of the 4 included trials [21, 22]. In 3 trials, HA was administered intra-articularly under landmark guidance [12, 13, 21], and 1 trial was performed under ultrasonography-guided injection [22]. The type of HA, number and dosage of HA injections, and postinjection follow-up duration varied widely between trials.

Two trials evaluated the added effect of intra-articular HA injections to conventional therapies [13, 21], and the other 2 compared intra-articular HA injection with conventional therapies [12, 22]. In the reviewed studies, the Visual Analogue Scale, the Verbal Numeric Scale, and the pain subscale of the Shoulder Pain and Disability Index were used as pain measures, and the Constant Score, the Shoulder Pain and Disability Index, and the Shoulder Disability Questionnaire were used as function/disability measures. Measurements for ROM included abduction, flexion, internal rotation, and external rotation.

### 3.2. Risk of Bias Assessments

The methodological quality of the included trials is shown in Table 3. The most common sources of potential bias were inadequate allocation generation and concealment. We did not combine the included trials using meta-analysis methods, because the number of included trials was small and heterogeneity existed in the study designs, interventions being compared (head-to-head comparison or as an added treatment), regimens, and preparations (molecular weight and viscosity) of HA, as well as in the outcomes of interest among the included trials.

### 3.3. HA versus Conventional Therapies: Pain, Function/Disability, and ROM

Two trials investigated the effectiveness of intra-articular HA injection as compared with conventional therapies [12, 22]. Calis et al. [12] divided participants into 4 groups and compared the effectiveness of intra-articular corticosteroid injection, intra-articular HA injection, physical therapy, and home exercise. Reported outcomes included shoulder pain (Visual Analogue Scale), function/disability (Constant Score), and ROM (passive) of the shoulder. The only significant difference observed in any measured outcome among the corticosteroid, HA, and physical therapy groups at 3 months after injection was significantly improved passive external rotation in the physical therapy group in comparison to the corticosteroid and HA groups. Park et al. [22] evaluated intra-articular HA administration with capsular distention (achieved by 0.5% lidocaine, 18 mL) in comparison to intra-articular corticosteroid injection. Reported outcomes included pain (Verbal Numeric Scale), function/disability (Shoulder Pain and Disability Index), and ROM (passive). The only significant difference that was obtained in any measured outcome at 10 weeks after injection was that passive external rotation was significantly improved in the HA (combined with capsular distention) group.

### 3.4. HA as Adjunct Treatment: Pain, Function/Disability, and ROM

Two trials investigated the effectiveness of intra-articular HA administration as an adjunct therapy in the treatment of patients with AC [13, 21]. Rovetta and Monteforte [21] compared combination therapy using intra-articular corticosteroid/HA administration and physical therapy to combination therapy using intra-articular corticosteroid injection and physical therapy. Reported outcomes included pain (Visual Analogue Scale) and ROM of the shoulder. No between-group comparison was performed, although both groups showed improvement of pain and ROM at 6 months after injection. They also observed that internal rotation was improved to a greater degree in the group that received intra-articular HA administration. Hsieh et al. [13] evaluated combination therapy using intra-articular HA injection and physical therapy in comparison to physical therapy alone. The reported outcomes included pain (pain subscale of the Shoulder Pain and Disability Index), function/disability (total scores of the Shoulder Pain and Disability Index and the Shoulder Disability Questionnaire), and ROM (passive and active). No significant difference was obtained in any measured outcome at 3 months after injection.

### 3.5. Adverse Events

Rovetta and Monteforte reported no major adverse events [21]. Park et al. reported that 12 out of 45 participants undergoing capsular distension combined with intra-articular HA injection experienced pain during the procedure, and the pain may be a result of the capsular distension procedure, the intra-articular HA injection, or both [22]. The adverse event data could not be obtained for the other 2 included trials [12, 13].

## 4. Discussion

Our review included 4 trials and provided a synthesis of the current evidence on intra-articular HA administration for the treatment of AC. Two trials investigated the effectiveness of intra-articular HA administration in comparison with conventional therapies, and the other 2 investigated the effectiveness of intra-articular HA administration as an adjunct therapy. The scopes of these reports are reflective of clinical practice. Intra-articular HA administration is not superior to conventional therapies (intra-articular corticosteroid administration and physical therapy) for the treatment of patients with AC, and the addition of intra-articular HA administration to conventional therapies is not expected to provide significant added benefits. Thus, the potential role of HA in the treatment of AC remains controversial.

In short-term follow-up assessments less than 3 months after administration, 2 of the included trials showed that the clinical effectiveness of intra-articular HA administration in terms of pain and shoulder function/disability was not superior to corticosteroid administration [12, 22], and this conclusion is also supported by a recent systematic review [11]. Intra-articular injections of either corticosteroids or HA reduce inflammation and pain in patients with AC and lead to functional improvement. The onset of the effects of corticosteroid administration is faster than HA administration, but the effects of HA may last longer than those of corticosteroids [10, 23]. Intra-articular administration of corticosteroids for the treatment of AC may be beneficial, although their effects may be relatively weak and only of short-term benefit [4, 24, 25]. Intra-articular administration of HA is more costly than corticosteroids for the treatment of AC, but fewer adverse events have been associated with HA administration [11]. In our review, the long-term effects (>6 months) of HA injection for the treatment of AC could not be ascertained because the included trials did not conduct long-term follow-up assessments.

Although the benefit of physical therapy in the treatment of AC is not confirmed by systematic reviews [26, 27], some clinical studies with lower grades of evidence have reported its benefits, and it is commonly used in AC treatment [3]. Calis et al. concluded that physical therapy provided clinical benefits superior to intra-articular HA and corticosteroid administrations [12]. Hsieh et al. also found significant improvements in patients treated with physical therapy [13]. Accordingly, physical therapy can be considered as a treatment option in patients with AC.

Rovetta and Monteforte administered corticosteroids alone or in combination with HA to treat AC [21]. Both treatment groups showed improvements in pain and ROM after 6 months of treatment, but the added benefit of HA to corticosteroid administration was not confirmed. Furthermore, Hsieh et al. showed that HA administration did not provide significant added benefits for patients with AC who were already receiving regular physical therapy [13]. Intra-articular HA administration for patients with AC who are already receiving regular conventional therapies should be carefully evaluated to avoid unnecessary medical expenditure.

Although the majority of the reviewed studies (3 of 4) used landmark-guided injection, the negative results of our review might not be attributable to the type of injection guidance (landmark or ultrasonography-guided). Lee et al. showed that the clinical effectiveness of ultrasonography-guided injection (1 initial corticosteroid dose, followed by 5 weekly doses of HA) for AC was not superior to landmark-guided injection after the first few weeks of treatment [20]. Similarly, ultrasonography guidance may improve the accuracy of corticosteroid injection for the treatment of shoulder pain, but clinical advantages of ultrasonography-guided injection over landmark-guided injection in terms of pain, function, ROM, and adverse events were not supported by the evidence [28, 29].

Our review differs from a previous review published in 2011 [11], because our review includes only randomized controlled trials, considers only studies with participants diagnosed with AC, reports shoulder pain outcomes in addition to function/disability and ROM, and focuses on the role of intra-articular HA administration compared to conventional therapies and role of HA administration as an added treatment. We also have included 2 trials that were published since 2011 [13, 22].

Several limitations of this review should be addressed. First, the small number of included trials limits the strength of the conclusions that were drawn. Second, the included trials are of variable methodological quality, which introduced the risk of bias (Table 3). Third, the participants recruited in our reviewed trials may differ with regard to the stage and severity of AC, and this may have limited our ability to measure beneficial effects of HA use in this review. Fourth, the majority of reviewed trials arranged follow-up assessments for 3–6 months after treatment, and thus the long-term effects of HA administration were not evaluated.

## 5. Conclusions

Intra-articular HA administration alone is not superior to conventional AC treatments, and the addition of intra-articular HA administration to conventional therapies does not provide significant added benefits. AC can be divided into 4 consecutive stages [3], and further randomized controlled studies involving large sample sizes and stage-based designs (analysis stratified by the stages of AC) are needed to verify the effectiveness of HA administration when used alone or in combination with other conventional therapies.

## Figures and Tables

**Table 1:** Detailed reasons for exclusion of individual studies.

Author	Year	Reasons for exclusion
Leardini et al. [16]	1988	Non-RCT (prospective uncontrolled study) Mixed diagnosis in the experimental group (OA, Duplay's disease, combined OA and Duplay's disease) Follow-up duration shorter than 1 month (11 days)

Itokazu and Matsunaga [17]	1995	Non-RCT (prospective uncontrolled study) Mixed diagnosis in the experimental group (subacromial bursitis, tendinitis, and capsulitis) Duration of symptoms shorter than 1 month in some subjects

Tamai and Yamato [15]	1997	Non-RCT (observational study)

Tamai et al. [18]	1999	Follow-up duration shorter than 1 month (7 days) Primary intention was not treatment of AC Participants in the AC group were the same as Tamai et al., 2004 [8]

Tamai et al. [8]	2004	Non-RCT (prospective uncontrolled study) Follow-up duration shorter than 1 month (1 week) Primary intention was not treatment of AC

Blaine et al. [19]	2008	Mixed diagnosis in the experimental group (OA, rotator cuff tear, and/or AC)

Lee et al. [20]	2009	Combined treatment (triamcinolone and hyaluronic acid injections) Primary intention was comparison of injection guidance

AC: adhesive capsulitis; OA: osteoarthritis; RCT: randomized controlled trial.

**Table 2:** Characteristics of the included trials.

Author	Year	*n*	Diagnostic tools	Mean age (years)	Gender (M/F)	Duration of symptoms (months)	Intervention	Total number of HA injection doses	Injection guidance	Injection site
Rovetta and Monteforte [21]	1998	30	History + PE + US	64.2	9/21	8.3	(1) HA + CS + PT: 20 mg HA + 20 mg triamcinolone + PT (4–12 weeks) (2) CS + PT: 20 mg triamcinolone + PT (4–12 weeks)	8 (15-day intervals in the first month and then monthly for 6 months)	Anatomy (posterior approach)	IA

Calis et al. [12]	2006	90 (95 shoulders)	History + PE	57.0	33/57	>1	(1) HA: 30 mg HA (2) CS: 40 mg triamcinolone (3) PT: 10 days (4) *Home exercises *	2 (weekly)	Anatomy (posterior approach)	IA

Hsieh et al. [13]	2012	63	History + PE	54.5	20/43	4.5	(1) HA + PT: 20 mg HA + PT (12 weeks) (2) PT: 12 weeks	3 (weekly)	Anatomy (posterior approach)	IA

Park et al. [22]	2013	90	History + PE + US	55.8	22/68	5.3	(1) HA: HA 20 mg + lidocaine (18 mL) (2) CS: triamcinolone 40 mg + lidocaine (4 mL)	3 (every 2 weeks)	US (posterior approach)	IA

CS: corticosteroid; F: female; HA: hyaluronic acid; IA: intra-articular; M: male; PE: physical examination; PT: physical therapy; US: ultrasonography.

**Table 3:** Methodological quality assessment of the included trials.

Author	Year	Allocation generation	Allocation concealment	Double blinding	Duration of follow-up^*^	Loss to follow-up (%)	Data analysis	Other bias
Rovetta and Monteforte [21]	1998	Unclear	Unclear	Unclear	6 months	0	ITT	

Calis et al. [12]	2006	Unclear	Unclear	Blinded assessor	3 months	0	ITT	

Hsieh et al. [13]	2012	Computer-generated random numbers	Unclear	Blinded assessor	3 months	10	PP	

Park et al. [22]	2013	Computer-generated random numbers	Adequate	Blinded participants and assessor	10 weeks	10	PP	Different volumes of lidocaine administered

^*^After first evaluation.

ITT: intention-to-treat; PP: per protocol.
